# CoV2-ID, a MIQE-compliant sub-20-min 5-plex RT-PCR assay targeting SARS-CoV-2 for the diagnosis of COVID-19

**DOI:** 10.1038/s41598-020-79233-x

**Published:** 2020-12-17

**Authors:** Stephen Bustin, Amy Coward, Garry Sadler, Louise Teare, Tania Nolan

**Affiliations:** 1 Medical Technology Research Centre, Faculty of Health, Education, Medicine and Social Care, Anglia Ruskin University, Chelmsford, Essex, CM1 1SQ UK; 2 Mid and South Essex NHS Foundation Trust, Broomfield Hospital, Chelmsford, Essex, CM1 7ET UK

**Keywords:** Biological techniques, Biotechnology, Molecular biology

## Abstract

Accurate, reliable and rapid detection of SARS-CoV-2 is essential not only for correct diagnosis of individual COVID-19 disease but also for the development of a rational strategy aimed at lifting confinement restrictions and preparing for possible recurrent waves of viral infections. We have used the MIQE guidelines to develop two versions of a unique five plex RT-qPCR test, termed CoV2-ID, that allows the detection of three viral target genes, a human internal control for confirming the presence of human cells in a sample and a control artificial RNA for quality assessment and potential quantification. Viral targets can be detected either individually with separate fluorophores or jointly using the same fluorophore, thus increasing the test’s reliability and sensitivity. It is robust, can consistently detect two copies of viral RNA, with a limit of detection of a single copy and can be completed in around 15 min. It was 100% sensitive and 100% specific when tested on 23 RNA samples extracted from COVID-19 positive patients and five COVID-19 negative patients. We also propose using multiple cycle fluorescence detection, rather than real-time PCR to reduce significantly the time taken to complete the assay as well as assuage the misunderstandings underlying the use of quantification cycles (Cq). Finally, we have designed an assay for the detection of the D614G mutation and show that all of the samples isolated in the Chelmsford, Essex area between mid-April and June 2020, have the mutant genotype whereas a sample originating in Australia was infected with the wild type genotype.

## Introduction

The emergence of severe acute respiratory syndrome coronavirus 2 (SARS-CoV-2) in 2019 as the causal agent of the COVID-19 disease^1,2^, and the ongoing pandemic has highlighted many of the inadequacies inherent in current diagnostic testing regimens^3^. As a result, the development of nucleic acid-based tests using different molecular approaches has been rapid. SARS-CoV-2 is currently identified using real-time reverse transcription (RT)-qPCR^4^, isothermal methodologies^5,6^ or CRISPR^7,8^. Isothermal approaches typically require more development and optimisation but have the advantage of speed and are more readily implemented into point-of-care systems^9^. PCR based assays have the advantage of simplicity of design, easier multiplexing potential and in most cases, greater sensitivity. Typical commercial tests use a one tube combined RT and amplification protocol, are carried out in fairly large volumes and use slow protocols with real time data acquisition that result in typical assay times of 1–1.5 h. They are broadly comparable, although their reported sensitivity of 500 viral copies per reaction^10^ is significantly lower than generally achievable using this technology, and assays differ significantly in the speed of their overall workflow^9^. Although a more recent publication describes a streamlined assay with a detection limit of 15 copies, this is based on experiments performed by spiking total human RNA with in vitro synthesised viral transcripts, and the total workflow time remains at 2 h^11^. Most SARS-CoV-2 RT-qPCR assays target two viral genes, and positive results are most commonly obtained through the hydrolysis of dual-labelled probes^12^. Targeting more than one viral gene is advisable as there are reports of significant false negative rates for SARS-CoV-2 RT-qPCR testing^13,14^. A potential challenge for any nucleic-acid based testing methods is the potential of the virus to mutate and an analysis of primer binding sites targeted by RT-qPCR assays has indeed shown that a high percentage are mutated^15^. A mutation in a reverse primer has been shown to affect the sensitivity of an RT-qPCR assay in use early in the pandemic^10^ and further mutations could lead to the occurrence of false negative results, particularly if the mutation occurs at the extreme 3′ end of the primers. Since false negative results could also be due to sampling difficulties, poor recovery of RNA, the presence of PCR inhibitors or human error, it is essential that the RT-qPCR assay itself is the least likely cause and includes controls to reveal as many areas of assay vulnerability as possible.

The principal aim of current SARS-CoV-2 diagnostic tests is to detect unambiguously the presence or absence of viral targets. Where viral load is not being determined, there is no requirement to quantify viral load and therefore no need to detect amplification products in real-time^16^. Instead, we have developed a multiple cycle fluorescence detection protocol that measures baseline fluorescence towards the beginning of the run and then records discrete increases after a specified number of cycles to detect target-dependent amplification, whilst at the same time saving the considerable time it takes to scan a plate at the end of each cycle.

**Figure 1:**
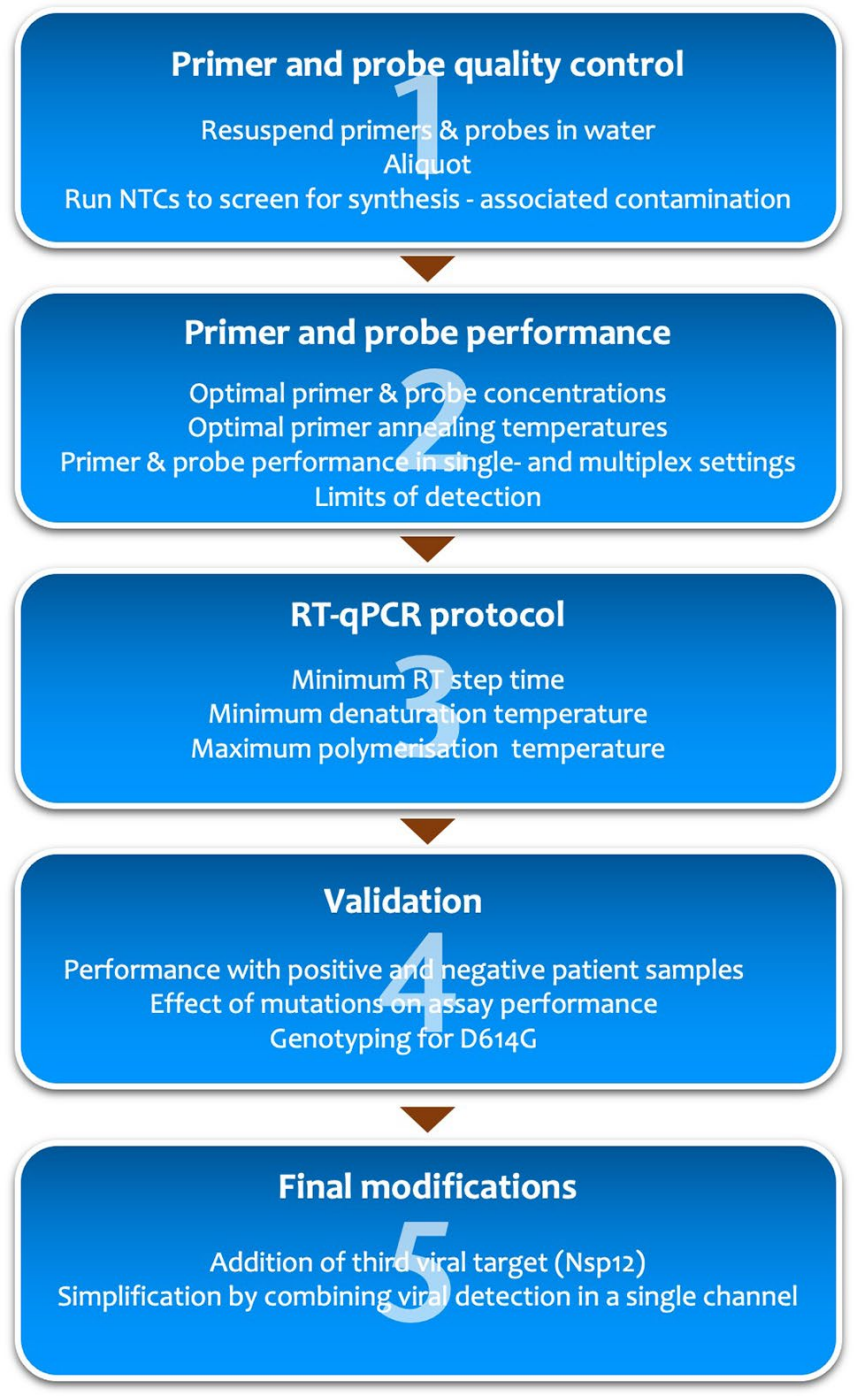
MIQE-compliant workflow used to characterise, optimise and validate the components that make up CoV2-ID assay.

Consequently, we set out to design an RT-qPCR test that can be used qualitatively or quantitatively, in addition to being sensitive, specific and fast. The Minimum Information for Publication of Quantitative Real-Time PCR Experiments (MIQE) guidelines^17^ were used as the basis for establishment of the workflow, resulting in CoV2-ID, a SARS-CoV-2-specific five plex RT-PCR test (Fig. 1).

This assay ensures specificity by targeting multiple SARS-CoV-2 genes (Nsp10, Nsp12 and N) in line with the WHO guidelines for the reliability of results, which require at least two genomic targets for diagnostic tests^18^, with neither primers and probes amplifying or detecting any other coronaviruses. The assay also includes a human control target (*JUN*) to confirm the presence of human cells and an extraction and inhibition control artificial sequence (EICAS) that can be used to monitor the performance of the assay and detect sample-induced reaction inhibition^19^. We have used droplet (dd) PCR to delineate quantification and detection limits of this assay, which can detect a single viral genomic target. Furthermore, having previously demonstrated that PCR reaction times can be significantly reduced by using shorter denaturation and polymerisation times and altering the denaturation and polymerisation temperatures^20^, we have developed a high-speed qPCR protocol that can be completed in around 15 min, depending on the instrument used. In addition, we demonstrate the feasibility of using a multiple cycle fluorescence detection protocol to reduce assay times even further.

## Materials and methods

The details of all reagents, plastic ware and instruments are listed in the supplementary data file, Table 1.

**Table 1:** Protocol for multiple cycle fluorescence detection.

Step	Reaction	Temperature (ºC)	Time (s)	Number of cycles
	Activation	95	30	–
Amplification 1	Denaturation	93	1	7
	Polymerisation	64	1	
Detection cycle 8	Denaturation	93	1	1
	Polymerisation	64	1	
Amplification 2	Denaturation	93	1	6
	Polymerisation	64	1	
Detection 15	Denaturation	93	1	1
	Polymerisation	64	1	
Amplification 3	Denaturation	93	1	4
	Polymerisation	64	1	
Detection cycle 20	Denaturation	93	1	1
	Polymerisation	64	1	
Amplification 4	Denaturation	93	1	4
	Polymerisation	64	1	
Detection cycle 25	Denaturation	93	1	1
	Polymerisation	64	1	
Amplification 5	Denaturation	93	1	4
	Polymerisation	64	1	
Detection cycle 30	Denaturation	93	1	1
	Polymerisation	64	1	
Amplification 6	Denaturation	93	1	4
	Polymerisation	64	1	
Detection cycle 35	Denaturation	93	1	1
	Polymerisation	64	1	

### Ethics statement

All methods were carried out in accordance with relevant guidelines and regulations. This study and experimental protocols were approved by the Research Ethics panel of the University's Faculty for Health, Education, Medicine and Social Care, reference HEMS-FREP 19/20/039. Analyses were performed on existing and anonymised RNA samples collected during standard diagnostic tests, with no clinical or epidemiological data available, apart from each reported quantification cycle (Cq). Consent was not required as the UK Human Tissue Authority has indicated that stored samples that have been taken for diagnosis and remain after the diagnostic procedure has been completed can be used in approved research, providing that all samples are anonymised to researchers.

### Sample collection, RNA extraction, selection and storage

Nasopharyngeal/nose and throat/throat samples were collected for routine SARS-CoV-2 testing by trained staff at Mid and South Essex NHS Foundation Trust Broomfield using a variety of swab types. RNA was extracted within 24 h of sample collection and eluted in a final volume of 50 µL RNase-free water. The standard protocol was modified to include an incubation at room temperature for 10 min in a Class 1 safety cabinet, before incubating at 56 °C for 10 min.

All RNA samples were stored at − 80 °C. Further validation of RNA sample quality was not possible due to diagnostic requirements, however subsequent testing with the EICAS assay (detailed below) provided a measure of inhibitory contamination^19^.

A total of 23 clinical RNA samples, in four batches (labelled A through D), testing positive for SARS-CoV-2 and one set of five samples testing negative at Broomfield Hospital were selected at random from those remaining after routine clinical tests. 5 µL aliquots were transported on ice for 3 miles from the hospital pathology laboratory to the Molecular Biology research laboratory (Anglia Ruskin University, UK). One of these samples had very high concentrations of viral RNA (A4) and so was diluted 1:100 with sterile RNase-free water and used for assay development. Eight further samples (A1 and C1-C7) were diluted 1:30 and used for optimisation, validation and comparative analyses. All samples were stored at − 80 °C until further use. Two control samples were included for development of the genotyping assay (kindly donated by J. Curry, LGC, UK). This first was RNA isolated from cell culture infected with Sars-CoV-2 which was isolated from a clinical sample at Westmead Hospital (New South Wales, Australia); COVID19 was confirmed using N1/N2 and E gene RT-qPCR) and the second, a synthetic control RNA (Twist Biosciences), which were included as wild type controls for D614G genotyping.

### Primers and probes

Human SARS-CoV-2 (NC_045512.2) and SARS-CoV-1 (NC_004718.3) reference sequences and a Bat SARS-like coronavirus genome (MG772933) were downloaded to the Allele ID 7 qPCR assay design software package and SARS-CoV-2 specific primers and probes were designed with manual adjustments aimed at maximising the analytical sensitivity and robustness of the assay. Three SARS-CoV-2 targets, Nsp10, N-gene and Nsp12, were chosen to accommodate sequence variabilities in primer or probe locations and minimise the likelihood of a 3′-mutation at primer binding sites reducing the reliability of the assay. *JUN* was chosen as a human extraction control to verify that human nucleic acid was present in the sample; it is intron-less, thus permitting detection of DNA and RNA, has low tissue-specificity and is highly expressed in proximal digestive tract (https://www.proteinatlas.org/ENSG00000177606-JUN/tissue). The specificity of primers, probes and amplicons was analysed in silico using Primer-BLAST (https://www.ncbi.nlm.nih.gov/tools/primer-blast/) and BLAST (https://blast.ncbi.nlm.nih.gov/Blast.cgi).

In addition, the human SARS-CoV-2 (NC_045512.2) genomic sequence was imported to the Beacon Designer 8.2 qPCR assay design software package and an LNA-based genotyping assay for discriminating the D614G mutation (A to G) was designed. Finally, two extraction and inhibition control artificial sequences (EICAS1 and 2) were designed so that they contained no matching sequences in published databases. EICAS1 is amplified using a bespoke primer set, whereas EICAS 2 was designed with terminal 5′ and 3′ sequences amplified by the *JUN* primers, to avoid potential primer interference during the multiplex PCR. EICAS1 and EICAS2 are detected by the same probe.

Upon receipt, all DNA oligonucleotides were resuspended in sterile RNase-free water at 100 µM and stored in aliquots at − 20 °C. The RNA oligonucleotides were diluted to 1 × 10^–9^ relative to the original stock with sterile RNase-free water and stored at − 80 °C.

### RT, qPCR and digital PCR

RNA extracted from clinical samples at a dedicated COVID-19 testing facility at the Broomfield Hospital Microbiology laboratory was tested for SARS-CoV-2 using Viasure’s single tube RT-qPCR protocol. RNA (5 µL neat sample) was tested in a final reaction volume of 20 µL, with a 15 min/45 °C RT step, 2-min polymerase activation step and 45 cycles of 10 s/95 °C denaturation and 50 s/60 °C annealing and polymerisation step using Biomolecular Systems’ Mic qPCR cyclers with manual threshold setting. Clinical laboratory testing protocols regarded samples as positive for SARS-CoV-2 if a Cq < 38 was recorded either for both ORF1ab and N gene targets or ORF1ab alone. The newly designed SARS-CoV-2 test described in this report, termed CoV2-ID, was validated on the 23 clinical samples (batches A–D) samples using a modified single tube RT-qPCR protocol (PrimeScript III, Takara and 1Step Go, PCRBio) with 1 µL RNA in a final reaction volume of 5 µL, a 5 min/50 °C RT step, 1 min polymerase activation and 40 cycles of 1 s/95 °C denaturation and 1 s/60 °C annealing and polymerisation. Absence of contamination was determined by running no template (NTC) and no RT (NRC) controls.

Subsequent one-step combined RT and qPCR reactions were carried out with 0.5 µL RNA, of sample-dependent, varying and unknown concentration, per 5 µL reaction. Where two tube reactions were carried out, 1 µL RNA was reverse transcribed in 10 µL with Superscript IV Vilo using random primers; EICAS cDNA synthesis was supplemented with specific reverse primer (R) at 10 nM final concentration. RT conditions were 5 min at 25 °C, 5 min at 50 °C and 5 min at 95 °C and cDNA was diluted into 20 µL of water, with 1–5 µL used for further analysis.

Primer optimisation was carried out using PrimeScript III by comparing Cqs using combinations of forward and reverse primers at 0.3, 0.6 and 1 µM and choosing the concentrations that recorded the lowest Cq. Probe optimisation was carried out by comparing Cqs obtained using 0.4 µM or 0.8 µM final probe concentration. Optimal annealing temperatures were determined by using SARS-CoV-2 cDNA and the temperature gradient option available on the BioRad CFX followed by melt curve analysis, together with SensiFast SYBR Green mix and optimal primer concentrations. Ideal annealing temperatures were identified as those resulting in the lowest Cq whilst retaining a single melt curve peak.

Minimum qPCR run times were established by preparing a single master mix, sufficient to carry out all experiments. The master mix consisted of SensiFast qPCR Probe mix, SARS-CoV-2 cDNA, and primers and probes specific for the targets and was kept on ice until required. 5 µL aliquots were subjected to qPCR for decreasing denaturation and polymerisation times, with 1 s for each step being the minimum possible on the qPCR instruments used.

The experiments aimed at reducing RT times were initially carried out as using PCRBio One Step RT-qPCR reagent, as the RT is supplied separately from the buffer. A 1× master mix of SARS-CoV-2 sample and EICAS RNA, CoV2-ID assay oligo blend (targeting Nsp10, N-gene, *JUN* and EICAS2) and RT-qPCR buffer was prepared and kept on ice. Immediately prior to each run, 10 µL of that master mix was placed in a microfuge tube, 1 µL of PCRBio RT was added and 2 × 5 µL were added to two wells of a 48 well qPCR plate. The plate was briefly spun and subjected to different RT times, with qPCR times kept constant. All experiments were repeated using PrimeScript III, but since this reagent is a single tube mixture of buffer and RT, a premix of RNAs, CoV2-ID and water was prepared, and 2× PrimeScript III mix was added just prior to each run.

RT-qPCR reactions were carried out on the following instruments: CFX (BioRad), Eco (PCRBio), Prime Pro (Techne) or Mic (BMS). Data were analysed using instrument software, Microsoft Excel for Mac v.16.38 and PRISM for Mac v.9.

Droplet digital PCR (ddPCR) reactions were set up as instructed in the operating guide using the QX200 droplet generator (BioRad), clear well semi-skirted 96 well plates, pierceable foil and PX1 PCR plate sealer (BioRad). Each reaction contained concentrations of cDNA corresponding to the ones used in parallel qPCR experiments, with the optimal primer and probe concentrations established for qPCR runs, which are slightly different from the recommended ones for primers (0.9 µM) and probe (0.25 µM). Following droplet generation, PCR reactions were carried out in 40 µL volumes on a C1000 Touch Thermal cycler (BioRad) using the standard program of 10 min enzyme activation at 95 °C, and 40 cycles of 30 s denaturation at 94 °C and 1 min annealing/polymerisation at 60 °C, with the ramp rate set to 2 °C/s. The droplets were analysed immediately on the QX200 reader. RT-ddPCR reactions added a 1 h reverse transcription at 45 °C at the start of the PCR protocol. Data were analysed using QuantaSoft Analysis Pro and QX Manager software.

### Multiple cycle fluorescence detection PCR

Multiple cycle fluorescence detection PCR assays were carried out using SensiFast in 5 µL volumes using the protocol shown in Table 1 on the CFX Connect instrument (BioRad). The first plate read after cycle 8 was used to establish a baseline fluorescence, thus allowing the calculation of fluorescence increases after cycles 15, 20, 25, 30 and 35. In each case the relative fluorescence change (∆F) was calculated by subtracting the cycle 8 fluorescence reading from the fluorescence readings after the respective cycles.

## Results

### Assay design and characteristics

In silico analysis by PrimerBlast and BLAST analyses of primers, probes and amplicons signified that all virus oligonucleotide sequences were specific for SARS-CoV-2 and that the assays were not complementary to any other coronavirus. Since the oligonucleotide manufacturer enclosed a cautionary note with primer and probe shipments indicating that they “could contain trace amounts of long oligo templates” specifying SARS-CoV-2 sequences, all panels were immediately assessed in qPCR assays without addition of template. None resulted in amplification signals, demonstrating that they were not contaminated with either SARS-CoV-2 or *JUN* templates. Assay details, oligonucleotide sequences, fluorophores, final optimised annealing temperatures (Ta) conditions and PCR efficiencies are shown in Table 2.

**Table 2:** Details of oligonucleotide sequences, fluorophores, optimal concentrations and annealing temperatures used for the detection of SARS-CoV-2 targeting Nsp10, N-gene, Nsp12, *JUN* and EICAS, collectively referred to as CoV2-ID.

Target	Target	Amplicon (bp)	Oligonucleotides (5′-3′)	Final conc. (µM)	Ta (°C)
SARS-CoV-2	Nsp10	85	F: GGATCAAGAATCCTTTGGTGG	1	64.0
			R: GTCACAAAATCCTTTAGGATTTGGA	1	
			Pr: FAM-CATCGTGTTGTCTGTACTGCCGTTGCC-Q	0.4	
	N-gene	88	F: GCTGCTAGACAGATTGAAC	1	60.5
			R: AGCAGATTTCTTAGTGACAGTTTG	1	
			Pr1:-TR-ATGTCTGGTAAAGGCCAACAACAACA-Q	0.4	
			Pr2: TR/FAM-TCTGGTAAAGGCCAACAACAACAAGG-Q	0.4	
	Nsp12	96	F: CATCCCTACTATAACTCAAATGAA	1	62.0
			R: GTCATAGTACTACAGATAGAGACAC	1	
			Pr: FAM-TGCAAAGAATAGAGCTCGCACCGT-Q	0.4	
	Mu-Nsp10	85	(10Mu) GGATCAAGAATC**T**TTTGGTGG	1	61.0
	Mu-N-gene	88	(NA) GCTGCTTGACAG**T**TTGAAC	1	62.0
			(NB) GCTGCTTGA**T**AGATTGAAC	1	60.3
			MuN: TR-T**T**TGGTAAAGGCCAACAACAACAAGG-Q	0.4	
	D614G mutation	104	F: CACCAGGAACAAATACTTC	1	
			R: CCAAGTAGGAGTAAGTTGA	1	
			WT: FAM-ctttAtcAggAtgTtaact-Q	0.4	
			Mu: HEX-ctttAtcAggGtgTtaact-Q	0.4	
mRNA control	*JUN*	88	F: CGCCTGATAATCCAGTCCA	1	60.5
			R: GCTCATCTGTCACGTTCTTG	1	
			Pr:- Cy5-CACATCACCACCACGCCGACC-Q	0.4	
EICAS1	–	58	F: AACAACCACACCAAAAC		
			R: GGAGGTTTTAGTTTGG		
			Pr: HEX-CACACAACACCAACAAAACCAAACA-Q		
	rArArCrArArCrCrArCrArCrCrArArArArCrCrArCrArCrArArCrArCrCrArArCrArArArArCrCrArArArCrArCrCrArArArCrUrArArArArCrCrUrCrC				
EICAS2	–	65	Pr: HEX-CACACAACACCAACAAAACCAAACA-Q		
	rCrGrCrCrUrGrArUrArArUrCrCrArGrUrCrCrArUrCrArCrArCrArArCrArCrCrArArCrArArArArCrCrArArArCrArCrArArGrArArCrGrUrGrArCrArGrArUrGrArGrC				

All oligonucleotides are listed in the 5′-3′ direction. Nucleotides that differ from the SARS-CoV-2 reference sequence, but have been detected at low frequency are shown in red. Oligonucleotides are based on the following accession numbers: Reference SARS-CoV-2: NC_045512; mutant SARS-CoV-2: MT412262 (F-primer 10Mu); MT607612/481905/496997/467255/467251 (F-primers NA and NB); MT506889/506904/506907 (Probe MuN); c-*JUN*: NM_002228.4.

The supplementary data file contains the detailed optimisation results for primer and probe concentrations (Table 2) and Tas (Table 3). All six assays resulted in efficient RT-qPCR assays, ranging from 94 to 103% (Table 2) with melt curves resulting in a single peak, indicating the amplification of a single amplicon (Supplementary Figure S1).

**Table 3:** Validation of 28 RNAs extracted from patients attending Broomfield Hospital between April and June 2020.

Sample	Date	Viasure			COV2-ID				Sansure			
		ORF1ab	N	IC	Nsp10	N	JUN	EICAS	ORF1ab	N	IC	D614G status
A1	24-04-2020	+ve	+ve	+ve	+ve	+ve	+ve	+ve	–	-	-	Mutant (G)
A2	29-04-2020	+ve	+ve	+ve	+ve	+ve	+ve	+ve	–	-	-	Mutant (G)
A3	30-04-2020	+ve	+ve	+ve	+ve	+ve	+ve	+ve	–	-	-	Mutant (G)
A4	01-05-2020	+ve	+ve	+ve	+ve	+ve	+ve	+ve	+ve	+ve	+ve	Mutant (G)
A5	01-05-2020	+ve	+ve	+ve	+ve	+ve	+ve	+ve	–	-	-	Mutant (G)
A6	03-05-2020	+ve	+ve	+ve	+ve	+ve	+ve	+ve	–	-	-	Mutant (G)
A7	03-05-2020	+ve	+ve	+ve	+ve	+ve	+ve	+ve	–	-	-	Mutant (G)
A8	03-05-2020	**−ve**	+ve	+ve	+ve	+ve	+ve	+ve	–	-	-	Mutant (G)
B1	14-04-2020	+ve	+ve	+ve	+ve	+ve	+ve	+ve	**−ve**	+ve	+ve	Mutant (G)
B2	26-04-2020	+ve	+ve	+ve	+ve	+ve	+ve	+ve	+ve	+ve	+ve	Mutant (G)
B3	26-04-2020	+ve	+ve	+ve	+ve	+ve	+ve	+ve	+ve	+ve	+ve	Mutant (G)
B4	26-04-2020	+ve	+ve	+ve	+ve	+ve	+ve	+ve	+ve	+ve	+ve	Mutant (G)
B5	27-04-2020	+ve	+ve	+ve	+ve	+ve	+ve	+ve	+ve	+ve	+ve	Mutant (G)
C1	14-04-2020	+ve	+ve	+ve	+ve	+ve	+ve	+ve	–	-	-	Mutant (G)
C2	15-04-2020	+ve	+ve	+ve	+ve	+ve	+ve	+ve	–	-	-	Mutant (G)
C3	15-04-2020	+ve	+ve	+ve	+ve	+ve	+ve	+ve	–	-	-	Mutant (G)
C4	15-04-2020	+ve	+ve	+ve	+ve	+ve	+ve	+ve	–	-	-	Mutant (G)
C5	17-04-2020	+ve	+ve	+ve	+ve	+ve	+ve	+ve	–	-	-	Mutant (G)
C6	17-04-2020	+ve	+ve	+ve	+ve	+ve	+ve	+ve	–	-	-	Mutant (G)
C7	19-04-2020	+ve	+ve	+ve	+ve	+ve	+ve	+ve	–	-	-	Mutant (G)
D1	13-06-2020	+ve	+ve	+ve	+ve	+ve	+ve	+ve	–	-	-	Mutant (G)
D2	27-04-2020	+ve	+ve	+ve	+ve	+ve	+ve	+ve	–	-	-	Mutant (G)
D3	13-06-2020	+ve	+ve	+ve	+ve	+ve	+ve	+ve	–	-	-	Mutant (G)
E1	N/A	**−ve**	**−ve**	+ve	**−ve**	**−ve**	+ve	+ve	**−ve**	**−ve**	+ve	N/A
E2	N/A	**−ve**	**−ve**	+ve	**−ve**	**−ve**	+ve	+ve	**−ve**	**−ve**	+ve	N/A
E3	N/A	**−ve**	**−ve**	+ve	**−ve**	**−ve**	+ve	+ve	–	-	-	N/A
E4	N/A	**−ve**	**−ve**	+ve	**−ve**	**−ve**	+ve	+ve	**−ve**	**−ve**	+ve	N/A
E5	N/A	**−ve**	**−ve**	+ve	**−ve**	**−ve**	+ve	+ve	**−ve**	**−ve**	+ve	N/A
n =	28											

The four positive (A-D) and single negative batches were collected at different times. Negative test results are highlighted in bold. The Viasure data were obtained at the hospital, and ten of the samples were screened using a commercial kit (Sansure). The result of the genotyping tests are also shown.

**Figure 2:**
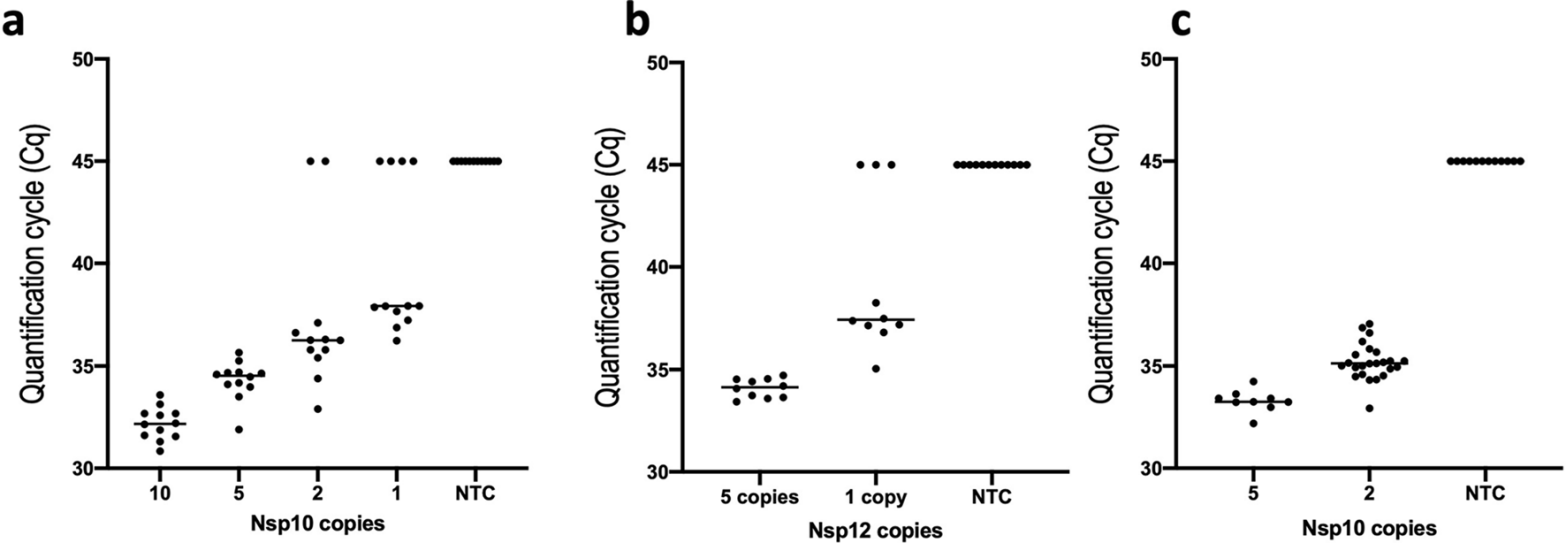
LOD for Nsp10 and 12. (**a**) Individual Cqs from 12 replicates of patient-derived samples containing nominal SARS-CoV-2 copies of 10, 5, 2 and 1 (determined by ddPCR), detected using the Nsp10 assay. (**b**) Cqs from 10 replicates with 5 or 1 copy of target obtained for Nsp12. (**c**) Cqs from repeat reaction of 10 replicates with 5 or 1 copy of target obtained for Nsp10.

A conservative limit of quantification was established based on results from ddPCR experiments using Nsp10. Results from a five-fold serial dilution series indicated that quantification was linear down to around 50 copies (Supplementary Figure S2a,b). In order to determine whether this was the approximate threshold of reliable and reproducible quantification, seven individual dilutions of the template were subjected to ddPCR assay. The results establish that this assay can reliably quantify 41 ± 12 copies of viral target (Supplementary Figure S2c,d), although this limit is lower if additional probes are used (see below). In order to translate this to an RT-qPCR limit of detection (LOD) for viral targets, the sample containing 50 copies was diluted further to nominal 10, 5, 2 and 1 copies and subjected to qPCR amplification using the Nsp10 assay. This resulted in the detection of 5 copies by 12/12 replicates and two and one copies by 10/12 and 8/12 replicates, respectively (Fig. 2a), with similar results obtained with Nsp12 (Fig. 2b). A repeat experiment using the dilution that had a predicted two-copy per reaction detected Nsp10 presence in 24/24 reactions (Fig. 2c). Underlying data are presented in supplementary data Table 4.

### Comparison of assay performance; individual or multiplex assays

**Figure 3:**
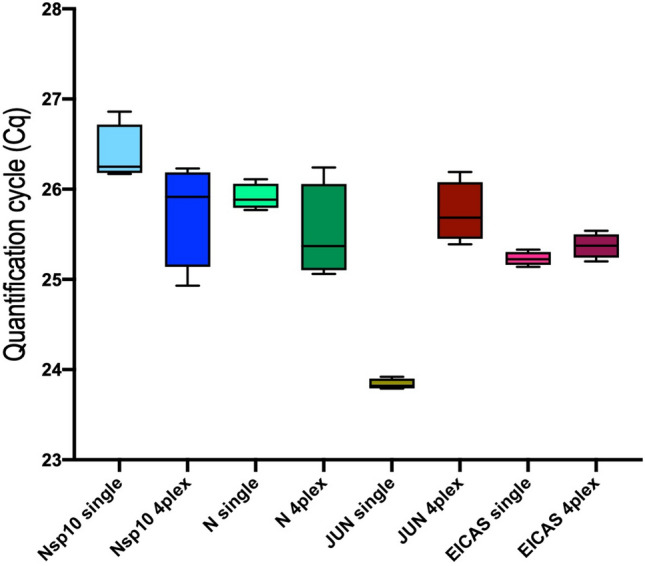
Comparison of Cqs obtained from single- and multiplex assays for the four panels making up CoV2-ID. The line through the box shows median Cqs and the whiskers denote minimum and maximum Cqs.

To reduce sample processing time, reduce reagent usage and increase throughput it was desirable to optimise the assays to run in multiplex. Two viral targets (Nsp10 (FAM) and N-gene (Texas Red), *JUN* (Cy-5) and EICAS2 (HEX) panels were combined to form the initial assay. Cq values obtained from assays run individually were compared with those obtained in the multiplex reaction. The assays perform equally well in both conditions (Fig. 3, supplementary data file Table 5) with only *JUN* amplification being approximately two cycles later in the multiplex reaction. This was solved by increasing the JUN primer concentration to 1.3 µM (Supplementary data file Table 5a).

The performance of the two EICAS assays and their effect on the amplification of the other markers was compared by carrying out four replicate multiplex RT-qPCR with either EICAS1 or EICAS2 as the internal control. Results were similar, suggesting that the inclusion of the additional primer set required for EICAS1 had no detectable adverse effect on assay performance (Supplementary data file Table 6).

### Assay validation

For validation of this test panel, 1 µL RNA was used per sample to reanalyse all 28 clinical samples and our results were 100% concordant (Table 3; Cqs are shown in supplementary data file Table 7).

For sample A8, Broomfield Hospital recorded discordant ORF1ab/N-gene results but these were positive for both markers when tested with our panel. Six positive and four negative samples were also tested using a commercial diagnostic kit (Sansure), with comparable results. In this case, the commercial kit did not detect one of the viral targets (ORF1ab) in sample B1, which was detected both at Bromfield Hospital and with our panel. There was significant correlation between Cqs recorded for Nsp10 and N-gene (r (95%CI) = 0.96 (0.89–0.98) as well as between Nsp 10 or N-gene and JUN (r = 0.73 (0.45–0.88) and 0.86 (0.68–0.94, respectively (Supplementary Figure S3 and supplementary data file, Table 7). An analysis of all clinical samples using the D614G genotyping assay revealed that all isolates harboured the A to G transition, characteristic of the more infectious phenotype, whereas the control clinical sample and Twist BioScience control 1 were both wild type, A, at this location (supplementary data file, Table 7).

Inclusion of the EICAS template in the assay panel permitted some analysis of the quality of RNA extracted from patient samples. If inhibitors of the RT or the PCR were present in the clinical samples, an increase in Cq was expected for the EICAS assay compared with no template control samples, analogous to the principle underlying the SPUD assay^19^. An analysis of the 28 samples revealed little, if any inhibition, with a median Cq of 27.07 (range 25.57–29.12) compared with the median Cq of 27.08 (range 27.54–26.91) recorded by no control samples (Supplementary data file, Table 7a).

The C to T transition at the − 9 position in the Nsp10 F primer binding site of isolate MT412262 did not impede the binding of the CoV2-ID F primer to mutant target (Supplementary Figure S4a). The reverse was also true, in that the mutant primer bound efficiently to the WT target (Supplementary Figure S4b). In each case the qPCR data were in broad agreement with the ddPCR results. Targets with mutations at positions − 10 and − 7 at the N-gene primer binding site were also efficiently amplified by the CoV2-ID F primer (Supplementary Figure S5c), as was the WT sequence by the two mutant primers (Supplementary Figure S5d). Since a mutation had been identified for three isolates (MT506889/506904/506907) at position 2 of the 5′-end of the N-gene probe, the effect of that mutation on the efficiency of amplicon detection by the N-gene probe was also investigated. Two specific probes were synthesised, one with (MuN) and one without (N-Pr2) the mutation at position 2 of the probe. Both gave virtually the same results (Supplementary Figure S5a), as did an alternative probe with WT sequences (Supplementary Figure S5b). The performance of both CoV2-ID and mutant primers with their respective templates was further analysed using an annealing temperature gradient analysis, which showed that the mutations had little effect on assay performance below 65 °C. Details of all underlying data for both qPCR and ddPCR results are listed in the supplementary data file Tabs 8, 8a, 8b and 8c.

### Conversion to a five plex assay and simplification

ddPCR data suggested that targeting two viral targets (Nsp10 and 12) using the same flurophore (FAM) increased the sensitivity of the assay by around 80% (Supplementary Figure S6a,b) and that there could be some benefit in a qPCR setting, especially with regards to further reducing the likelihood of a false negative result (Supplementary Figure S6c,d with well statistics and Cqs listed in supplementary data file Table 9).

To test this concept, Nsp12 was added as a third viral target to the four plex CoV2-ID assay, making it a five plex, with two of the viral targets being detected on the FAM channel. The results confirmed a reduction in Cq in the FAM channel, without affecting any of the other results (Supplementary Figure S7, with underlying data in supplementary data file Table 10). Performance of the four- and five plex assays was further assessed in four additional patient samples and the results indicated that they performed comparably, with the viral targets being detected earlier in the five plex assay (Supplementary Figure S8, with underlying data in supplementary data file Table 11). Finally, in order to determine whether the sensitivity of the assay could be increased further, the five plex assay was modified so that all three viral targets (Nsp10, Nsp12 and N-gene) were detected with FAM-labelled probes. Both qPCR (Figure S9a,b) and ddPCR (Supplementary Figure S9c,d) data revealed that there was indeed a further increase in sensitivity (data in supplementary data file Table 12).

### Development of rapid cycling conditions

**Figure 4:**
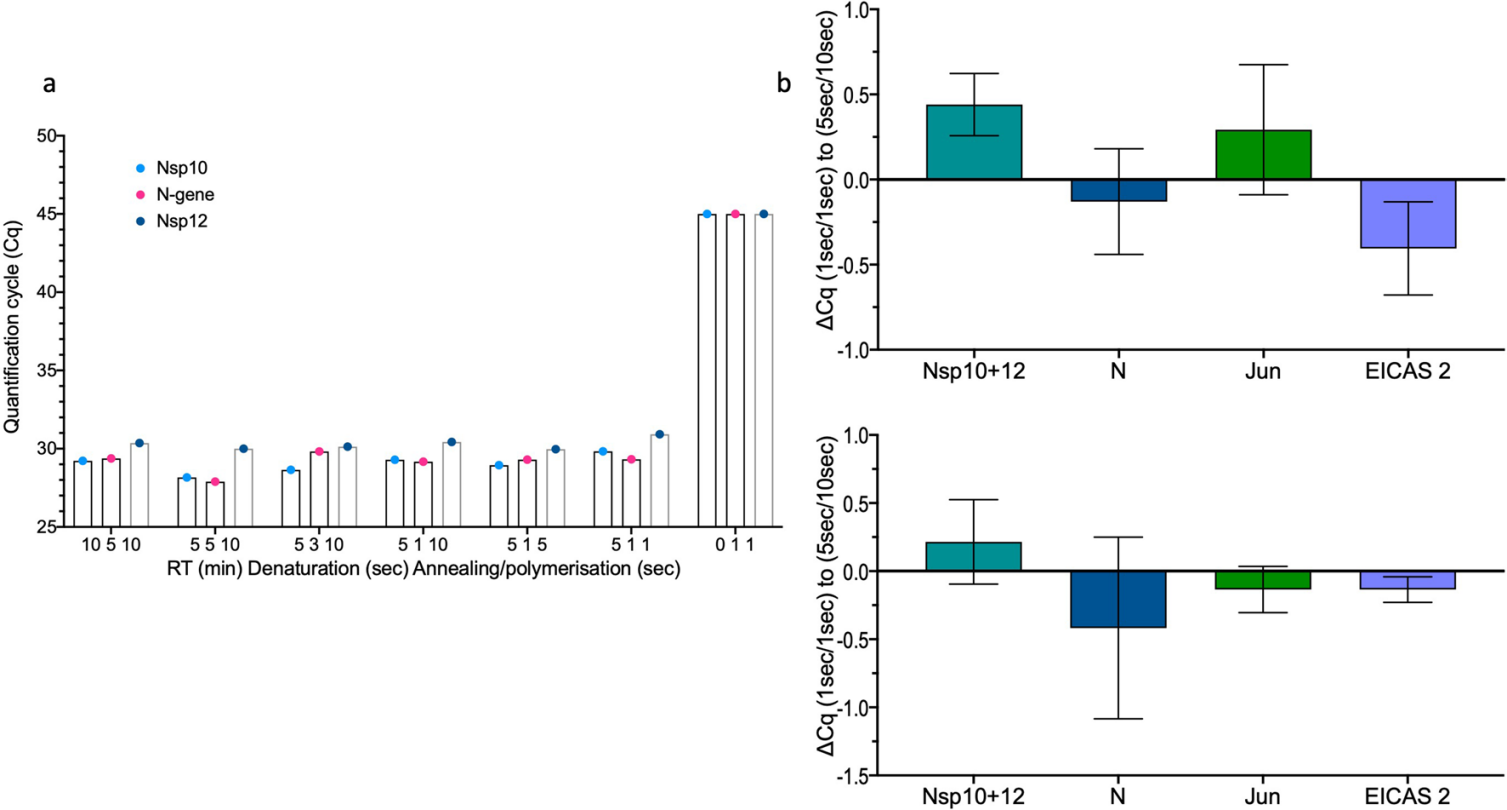
Reduction in qPCR times. (**a**) Cqs obtained for Nsp10, 12 and N-gene by reduction of qPCR times from 5 s denaturation and 10 s annealing polymerisation to 1 s each. (**b**) Comparison of the initial (5 s/10 s) and final (1 s/1 s) denaturation/annealing and polymerisation qPCR protocols. The assays were run in duplicate, with the plots showing the ∆Cqs between the longer and shorter timings.

In order to further improve the potential throughput of the assay in a diagnostic setting, the ability of the assay to perform adequately under short RT times and fast PCR conditions was tested. Baseline Cq data from the initial conditions of 10 min RT, 5 s denaturation and 10 s polymerisation were obtained for all three viral targets. Then RT time was reduced to 5 min, followed by stepwise reduction of both denaturation and polymerisation times to 1 s each, although in practice, the annealing/polymerisation step took around 6 s, as the fluorescence scanning required around five seconds. There was no decline in the performance of the assay, but the run times were reduced from 33 min 40 s to 20 min (Fig. 4a, with data in supplementary data file Table 13). The initial 5 s/10 s and final 1 s/1 s conditions were applied to replicate five plex assays, and the results confirmed that all panels could be run using this protocol (Fig. 4b; with data in supplementary data file Table 13b).

The next aim was to try and reduce run times by further reducing the RT times. The results shown in supplementary Figure S10 (underlying Cqs are in supplementary data file, Table 14) suggested that a 1-min RT step resulted in Cqs similar to the 5-min RT reaction, reducing run times to 16 min.

Reductions in run times could also be achieved on instruments not designed to run as fast as the PCRMax/Techne, as shown for the BioRad CFX. Here the reduction in RT time from 10 to 1 min and cycling times from 95 °C/5 s and 60 °C/20 s, to 1 min RT and 1 s each at 95 °C and 60 °C reduced the run time from 58 to 32 min. The Cqs from seven targets present at a wide range of concentrations were compared and there was very little difference. Indeed, most of the targets recorded slightly lower Cqs with the fast run (supplementary data file, Table 14a).

**Figure 5:**
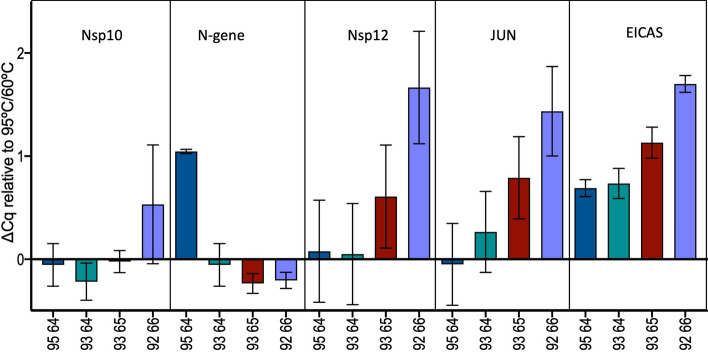
Reduction in denaturation and polymerisation times. The ∆Cqs for each of the five targets are plotted for each of the different temperatures used to carry out the PCR reactions.

Since the cooling step is the slowest part of the PCR cycle in block-based qPCR instruments, reducing the temperature gap between denaturation and annealing/polymerisation temperatures should further reduce run times. Following an initial calibration run with a 1 min RT step followed by 1 s 95 °C denaturation and 60 °C annealing/polymerisation steps, denaturation temperatures were reduced and annealing/polymerisation temperatures were increased. Even without further modifications to primer or enzyme concentrations, the small differences in Cq (Fig. 5, with Cqs in supplementary data file, Table 15) indicated that this would be a potential method to reduce reaction times further, in this case from 16 to 14 min 11 s.

### Multiple cycle fluorescence detection

**Figure 6:**
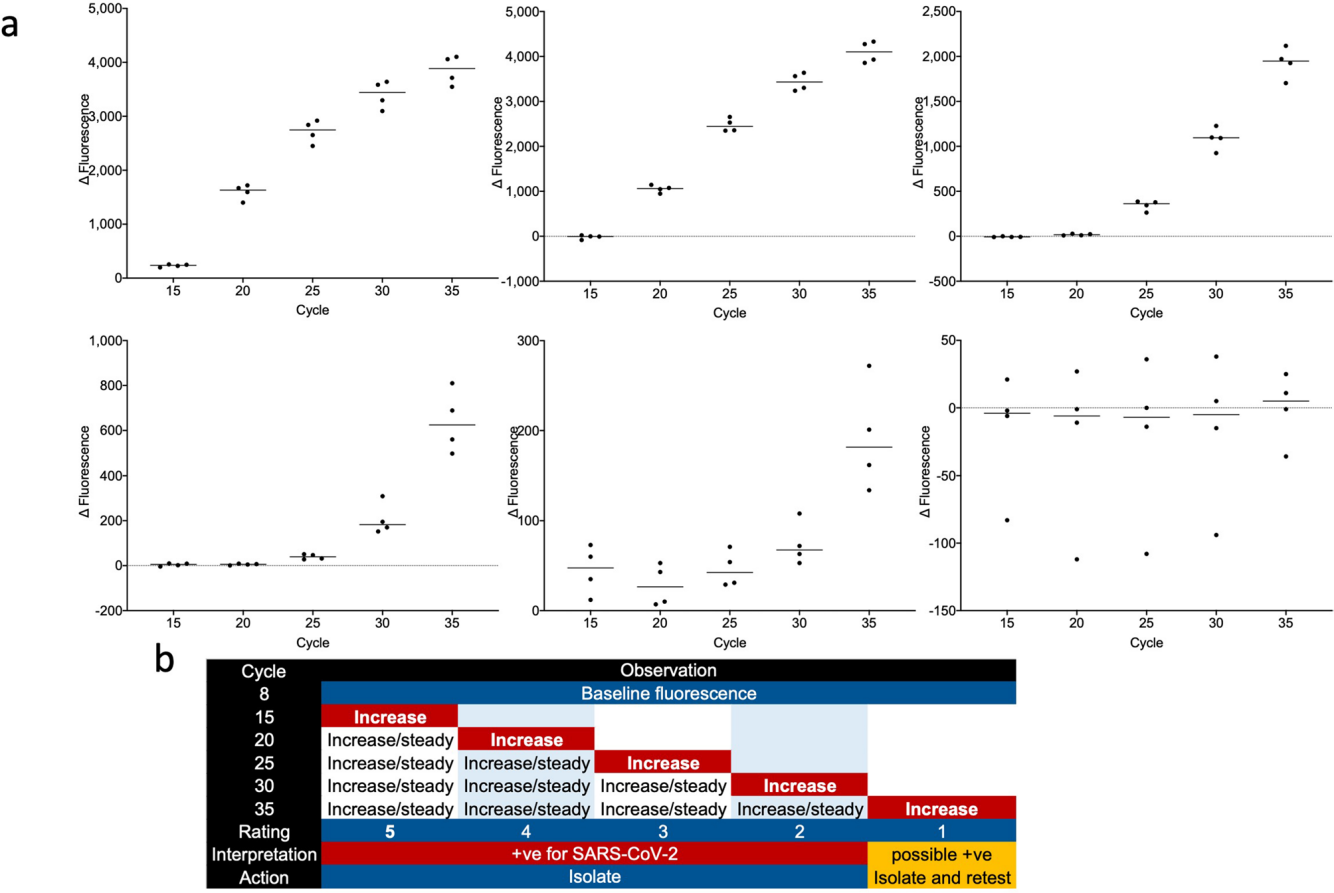
Multiple cycle fluorescence detection PCR results. (**a**) Four replicates were assayed for five samples containing different concentrations of Nsp12 amplicons, together with a NTC. Fluorescence data were collected at cycles 8, 15, 20, 25, 30 and 35 and for each replicate the fluorescence recorded at cycle 5 was subtracted from the fluorescence recorded at each subsequent fluorescence collection cycle. The difference in fluorescence was plotted for each cycle, with the horizontal bar indicating the median fluorescence. The run took just over 22 min to complete. (**b**) Algorithm incorporating the fluorescence data into a diagnostic assessment tool, obviating the need to deal with Cqs and cut-offs.

We have developed a multiple cycle fluorescence detection (MCFD) protocol linked to a 5-level rating algorithm. This resulted in faster run times and permitted the inclusion of the quantitative information inherent in qPCR without the confusion surrounding the use of quantification cycles. The feasibility of using this method rather than real-time detection was tested by comparing the performance of the two approaches using the same master mixes. As the PCRMax/Techne software does not permit a PCR run to be carried out without collecting fluorescence data, the experiment was carried out on the BioRad CFX. Whereas the standard qPCR run took 43 min to complete, the MCFD run took just over 22 min. The results for five different concentrations of viral target, together with the NTC control are shown in Fig. 6a, with the proposed algorithm in Fig. 6b. All underlying MCFD data are shown in supplementary data file Table 16.

### Quantification potential

The inclusion of ddPCR quantified, internal EICAS, facilitates an indirect measurement of copy number, thus allows this RNA to function both as a measure of quality control, as well as an assessment of viral load.

The same quantity of Nsp10 target was detected using qPCR as well as digital PCR. The results shown in Supplementary Figure S11a demonstrate how the reported Cq depends on the threshold setting, which is subjectively set by the operator or automatically determined by a software algorithm that can vary between runs and instruments. This interferes with accurate quantitative reporting of SARS-CoV-2 viral loads, as the highest and lowest threshold-dependent Cq recorded in that run varies by 8.7, ie corresponds to a 400-fold difference. In contrast, the copy numbers calculated using the ddPCR platform showed little variation, recording an average copy number of 1163 ± 61 (Supplementary Figure S11b) Underlying data are shown in supplementary data file Table 17.

## Discussion

The COVID-19 pandemic, caused by the new SARS-CoV-2 virus, has led to the development of a wide range of diagnostic assays, many of which are RT-PCR based, utilise real time detection and report a Cq value to indicate presence or absence of the virus. However, it has become increasingly clear that there are significant shortcomings in the use and interpretation of many of these assays for testing and monitoring populations for viral spread. This has resulted in some confusion as to whether these diagnostic assays are capable of adequately addressing their three main functions: First, to identify patients presenting with symptoms consistent with COVID19 as SARS-CoV-2 positive or negative. Second, to provide a meaningful assessment of viral load, given that Cqs are subjective and not sufficiently reproducible or robust to allow an appraisal of the validity of marginal results, i.e. those around cycle 35. Third, to monitor the spread of virus using screening programmes of populations and environmental samples, allowing that a high percentage of those infected remain asymptomatic.

In general, the most critical features of a diagnostic assay are high specificity and sensitivity, with reliability, speed, and ease of use also being highly desirable. In addition, where large numbers of samples are processed, cost saving on reagents is a serious consideration. Well-designed RT-qPCR assays certainly fulfil the first two criteria and have the potential to meet the remaining ones. Each of the three scenarios above requires a subtle variation on assay design and application. In the first case, the assay must be highly specific, reliable, sensitive and rapid. At this stage a binary positive or negative readout is sufficient. However, in the second scenario where therapy may be informed by an indication of viral load, the assay requires the same features with the addition of a quantitative assessment. Finally, widespread screening protocols benefit from low cost, high throughput and require simple binary readout without the absolute requirement to reduce time. The assay described in this communication, termed CoV2-ID, has been developed to be adapted to any of the situations described.

CoV-2-ID offers maximum sensitivity by targeting three viral targets and detecting their presence using the same fluorophore, building on a previous report, which used two hydrolysis probes to increase the sensitivity of a SARS-CoV-2 assay^21^. This is appropriate for a diagnostic test, which does not need to distinguish between different viral targets and increases the sensitivity by around threefold, thus improving on currently used commercial assays.

Whilst it may be desirable to measure viral load in patients exhibiting COVID-19, there is considerable misunderstanding with regards to the quantitative interpretation of diagnostic RT-qPCR test results. These are routinely recorded as stark Cqs, which can be rather misleading. When RT-qPCR is used to quantify gene expression, Cqs are normalised against reference genes, with amplification efficiencies determined by dilution curves^22^ and meaningful quantification dependent on efficiencies being similar between targets of interest and reference genes. Importantly, if not normalised, Cq values are subject to inherent inter-run variation^23^ and are operator, reagent- and instrument-dependent. Huge variations in Cq value ranges of up to 21 Cqs, the equivalent of a 2 × 10^6^-fold difference, have been reported for the same clinical virology run controls run in different laboratories^24^. Consequently, results are not readily comparable when carried out using different master mixes, instruments and analysis criteria and should not be used without appropriate calibration standards^17^. Since Cq values are affected by numerous parameters, not least by operator intervention with regards to threshold settings, as shown in Supplementary Figure S11, they must not be used quantitatively and should not be used to determine viral loads. Hence, in the absence of certified controls or even validated standards, the inclusion of the EICAS RNA control template, quantified by ddPCR, allows not just monitoring of the RT-qPCR reaction but also quantification of viral load. It must be stressed, however, that any quantitative data will still be laboratory-specific, and that use of the EICAS must be validated separately and repeatedly in different laboratories. The EICAS serves additional control functions in the assay: It provides an indication of potential inhibitors of the RT or PCR within the sample and has the potential to serve as an RNA extraction control. The addition of an intron less human gene control permits screening of samples for nucleic acid content regardless of whether DNase is used during sample preparation.

The single tube reaction format, with low volumes and all reagents premixed, results in a simple workflow and we have minimised RT and qPCR times^20^ to generate an assay that can be run in less than 20 min on a suitable instrument. Furthermore, since it is useful to run a diagnostic test as quickly as possible, we have combined endpoint and real-time analysis by measuring fluorescence towards the beginning of the run and then monitoring any increase at defined intervals thereafter. This permits the detection of target-dependent amplification, whilst at the same time saving the considerable time it takes to scan a plate at the end of each cycle. Our data demonstrate that this results in fast run times, even on an instrument such as the BioRad CFX, which is not optimised for maximum speed. The incorporation of defined fluorescence detection cycles into a reporting algorithm also has the advantage of allowing the quantitative aspect of qPCR to be used to generate a viral load determination, without resorting to the use of a subjective Cq or controversial cut-off point. This algorithm associates increases in fluorescence with a 5-level ratings system that can be used to inform further action, with any patient testing at level 1 being retested immediately. Importantly, this protocol can be applied to most conventional qPCR instruments, in contrast to a recent report that requires specialist instrumentation^25^.

It is widely accepted that SARS-CoV-2 mutations are arising constantly, although there is still no evidence for the evolution of distinct phenotypes in SARS-CoV-2^26^. Nonetheless, a SARS-CoV-2 variant carrying the Spike protein amino acid change D614G has replaced the original D614A variant in many locations^27–29^. This mutation increases infectivity^30,31^ and may increase the severity in infected individuals^32^, although it remains unclear what the impact of the mutation on transmission, disease, and vaccine and therapeutic development is^33^. These data certainly support the finding that this variant is ubiquitous, as all of the UK isolates tested back as early as 14th April carry the D614G mutation.

Although human coronaviruses harbour a proofreading exoribonuclease, a number of location-specific mutations have been identified in the genome of SARS-CoV-2 that result in potential mismatches with all published primer and probe sequences (https://covid19.edgebioinformatics.org/#/assayValidation)^15^. Hence, it is important to continue monitoring any assay, so as to sustain routine scrutiny of sequence mutations in primer and, to a lesser degree, probe binding regions of the viral genome, as recommended by the American Society for Microbiology COVID-19 International Summit^34^. CoV2-ID targets three viral genes, as this allows scope for the test to remain accurate even if mutations arise at the 3′-ends of the primers that could result in false-negative results. Our analysis of the effects of mutations in primers suggest that their impact is limited, as long as they are not at the 3′-ends of the primers. Variants with C to T transitions at positions − 9 and − 10 and an A to T transversion at position − 7 of the N-gene present a frequency of 0.006%^35^ displayed no difference in sensitivity compared to the WT assay. Two mutations within the N-gene probe binding site, one a G to T transversion at 5′-end nucleotide 3, the other a C to T transition at nucleotide 5 with respective frequencies of 0.053% and 0.029% also had no discernible effect on sensitivity.

CoV2-ID is presented as an adaptable assay that can be applied to all SARS-CoV-2 detection and quantification applications. In presenting the development of this assay we also sought to provide maximum transparency for the verification process, effect of protocol variations and the requirement for adequate standards and controls in order for the assay to be reliable and robust. The current pandemic has revealed shortcomings in global response procedures and it is essential that public health institutes, regulatory bodies and standards organisations adopt a shared set of guidelines, protocols and standards that allow a common and meaningful interpretation of any emerging molecular testing regimen.

In conclusion, we have used the MIQE guidelines to design, develop, optimise and validate CoV2-ID, an enhanced, value-added RT-qPCR assay specific for SARS-CoV-2. It is robust, sensitive and is optimised for a rapid protocol, providing the opportunity for high throughput, multiplex viral detection with the potential to quantify viral load. Its design minimises the likelihood of assay failure causing false negative test results and its robustness provides a promise for its further development as an extreme PCR assay for use with point of care devices.

## Supplementary Information

Supplementary Information 1.

Supplementary Information 2.
