# Supplementary material for: CoV2-ID, a MIQE-compliant sub-20-min 5-plex RT-PCR assay targeting SARS-CoV-2 for the diagnosis of COVID-19
Source: Sci Rep. 2020 Dec 17;10:22214. doi: 10.1038/s41598-020-79233-x (PMC7747624; doi:10.1038/s41598-020-79233-x)
Supplement: Supplementary file 1 — Supplementary Information 1. [file 41598_2020_79233_MOESM1_ESM.pdf]

CoV2-ID, a MIQE-compliant sub-20-minute 5-plex RT-PCR assay targeting SARS-CoV-2 for the diagnosis of COVID-19

Stephen Bustin, Amy Coward, Garry Sadler, Louise Teare, Tania Nolan

Supplementary Figures 1-11

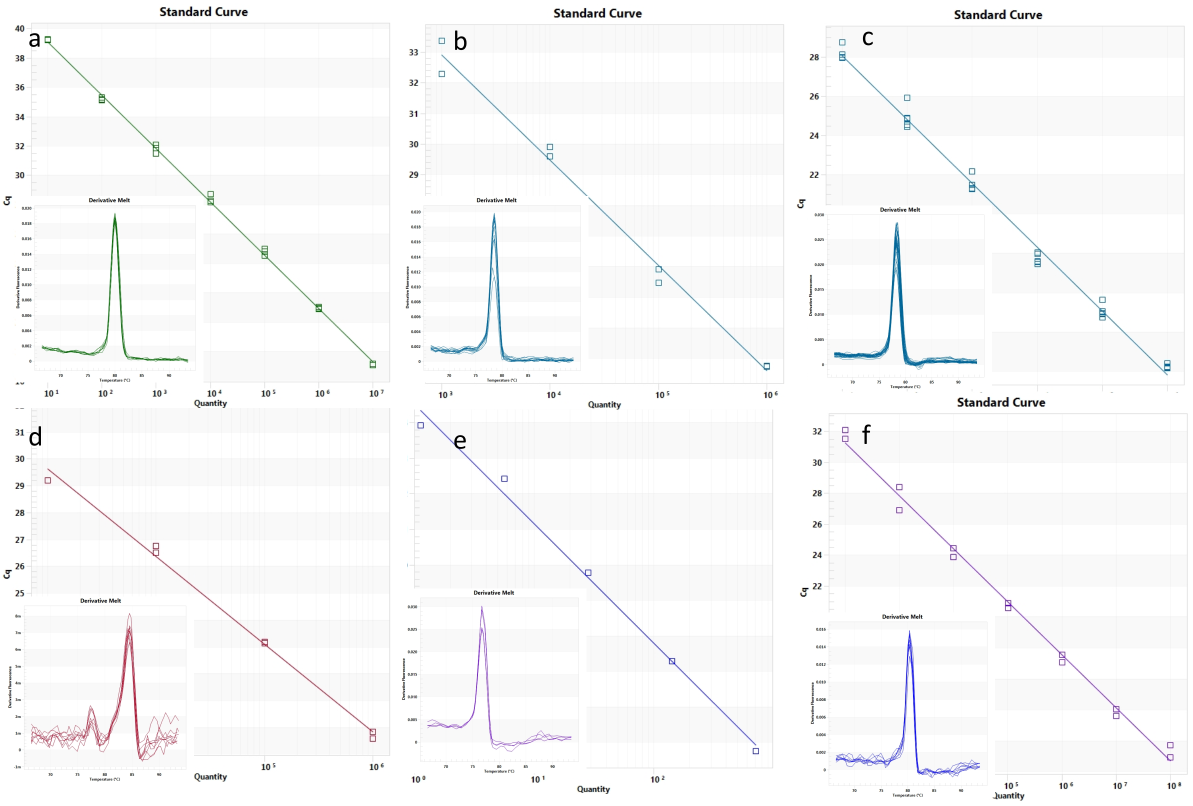

Figure S1. Standard curves and derivative melt plots for CoV2-ID assay components and the two EICAS amplicons. a. Nsp10 (y=-3.49x+39.96). b. N-gene (y=-3.43x+43.2). c. Nsp12 (y=-3.35x+37.82). d. c-JUN (y=-3.26x+39.4). e. EICAS1 (y=-3.36x+34.52). f. EICAS2 (y=-3.43x+38.12).

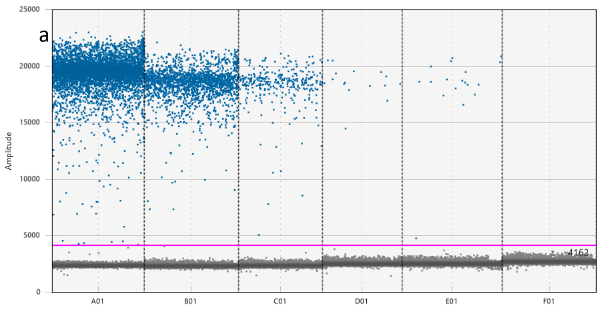

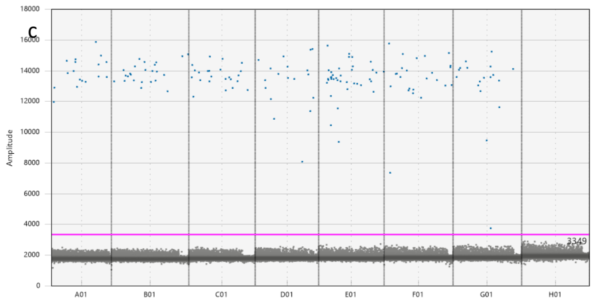

b

Sample	Copies/20µLWell	Accepted Droplets	Positives	Negatives
A01	22535	8,336	5137	3,199
B01	5712	8,550	1843	6,707
C01	1022	7,601	323	7,278
D01	52	7,225	16	7,209
E01	47	9,065	18	9,047
F01	0	8,529	0	8,529

d

Sample	Copies/20µLWell	Accepted Droplets	Positives	Negatives
A01	34	11,786	17	11,769
B01	37	15,251	24	15,227
C01	41	13,074	23	13,051
D01	34	12,509	18	12,491
E01	68	12,888	37	12,851
F01	42	13,586	24	13,562
G01	31	13,516	18	13,498
H01	0	13,366	0	13,366

Figure S2. ddPCR determination of LOD for Nsp10. a. 1D amplitude view of five-fold serial dilutions, with positive droplets above the threshold (pink line). b. Well data statistics. The sample in well F01 is the NTC. c. 1D amplitude view of repeat dilutions to a nominal 50 copies. d. Well data statistics. The sample in well H01 is the NTC.

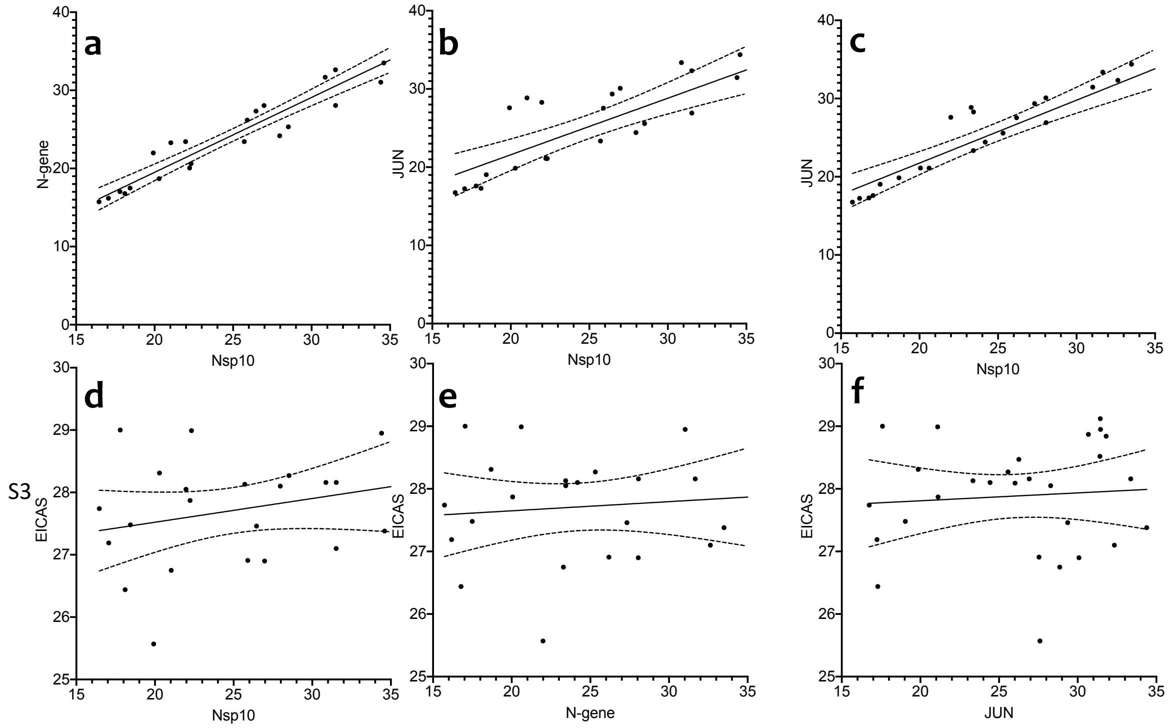

Figure S3. Linear regression analyses. The tabular results are listed in the supplemental results data file tab 7 a. Nsp10 vs N-gene. b. Nsp10 vs JUN. c. N-gene vs JUN. d, Nsp10 vs EICAS, e. N-gene vs EICVAS. f. JUN vs EICAS.

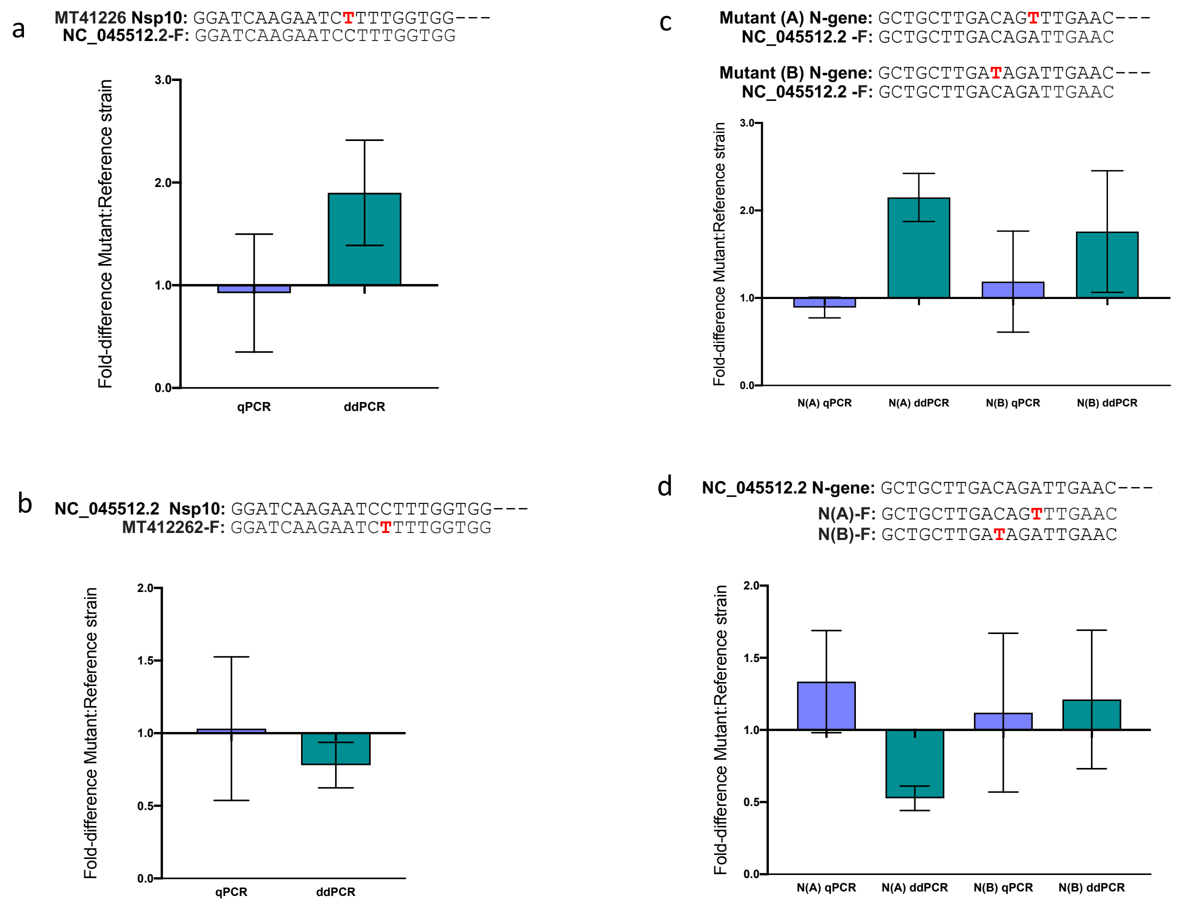

Figure S4. Analysis of mutation in Nsp10 and N-gene F primers. a. Position of C to T transition in strain MT412262 at F primer position -9, shown in red. For the qPCR results, the difference between Cqs obtained for amplification with WT and mutant primers (ΔCq) from four experiments were converted to a fold-difference (2^ΔCq) and plotted, with error bars showing the standard deviation (supplementary data file tab 8). For the ddPCRc results, the difference in calculated copies/well of WT and mutant from two experiments were converted to a fold-difference (WT/Mu) and plotted with the standard deviation (supplementary data file tab 8a). b. Position of C to T transition in WT sequence, shown in red on the mutant F primer. D. The fold-differences were obtained as described above. c. Positions of A to T and C to A transversions in mutant templates at N-gene F primer positions -7 and -10, shown in red. The plot of the fold-differences was generated as described above d. Positions of mutated bases on primers N(A)-F and N(B)-F, aligned with WT template and shown in red. The plot of the fold-differences was generated as described above. qPCR and ddPCR data are shown in supplementary data file tabs 8 and 8a, respectively.

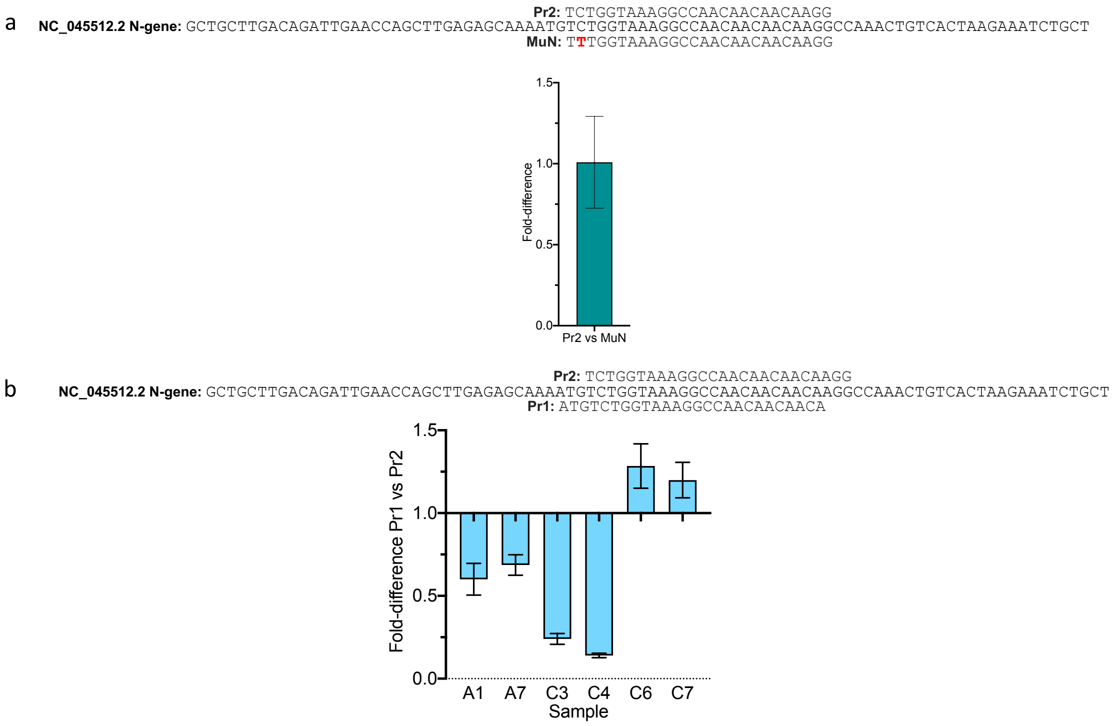

Figure S5. Analysis of mutated and alternative probes for N-gene. a. Positions of C to T transition at the 5'-end of the MuN probe shown in red. The plot shows the fold-difference obtained when Pr2 or MuN probes were used to detect wild type SARS-CoV-2 amplicon. The error bar indicates the standard deviation from two experiments carried out in duplicate (supplementary data file tab 8b). b. Positions on the N-gene amplicon of the two probes used to detect the N-gene. D. The plot shows the fold-difference obtained when Pr2 or MuN probes were used to detect RNA extracted from six clinical samples (supplementary data file tab 8b).

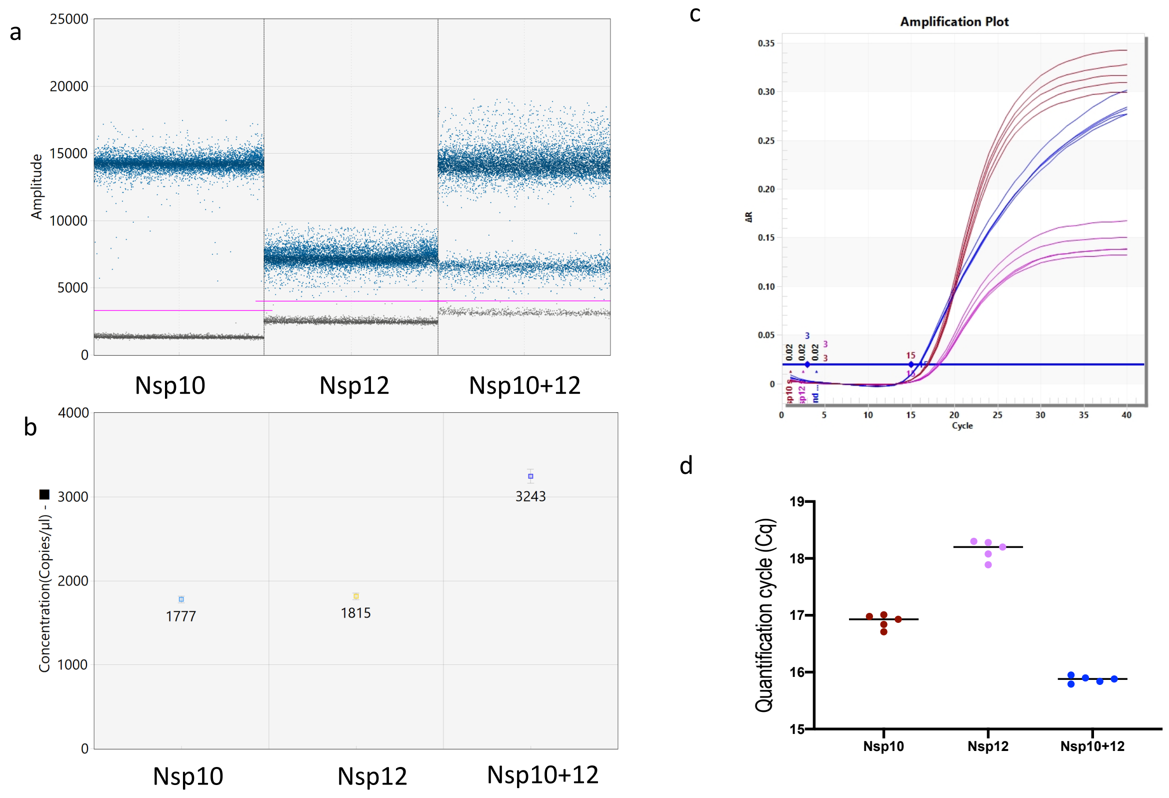

Figure S6. Effect of using two FAM-labelled probes to detect viral targets. a. ddPCR result showing the 1D amplitude of Nsp10 and Nsp12 either in separate wells or together. b. Copy numbers of targets detected by Nsp10, Nsp12 and both probes calculated from the results shown above. c. The same samples amplified in a qPCR assay as four replicate reactions. d. Cqs of the three qPCR assay conditions plotted. The horizontal bar indicates median Cqs.

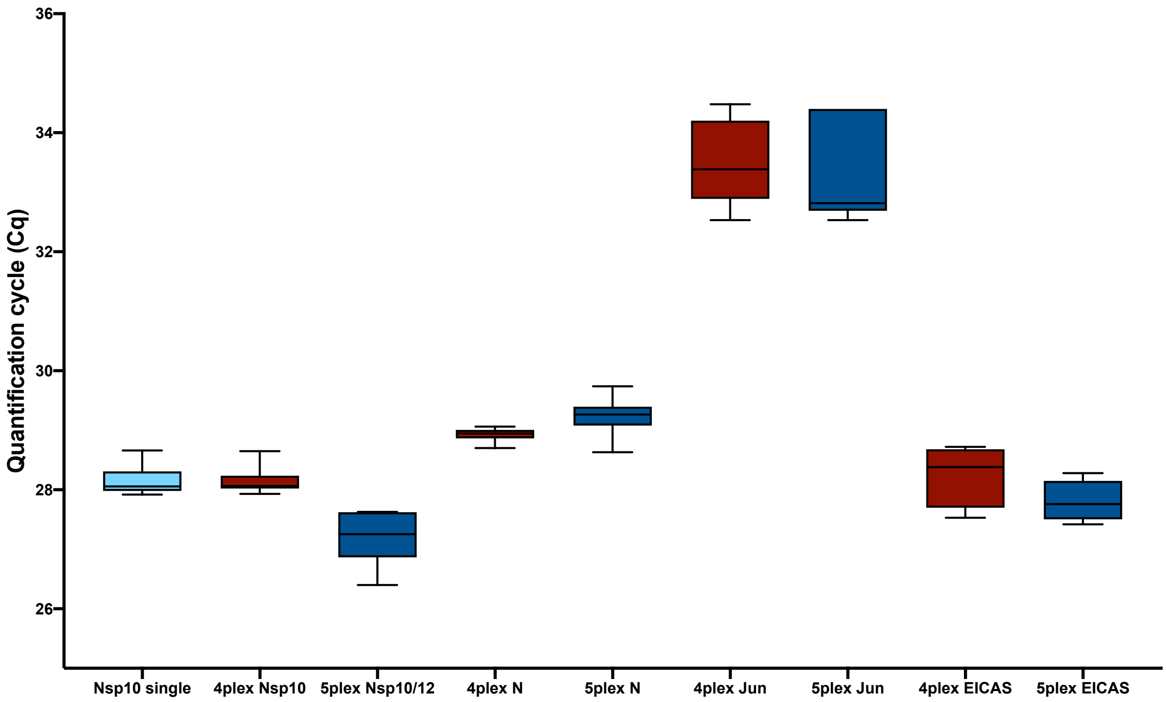

Figure S7. Effect of adding additional target (Nsp12) to CoV2-ID panel. Compared with the single (light blue) and 4plex (brown) assays, the 5plex assay (blue) records lower Cqs, with similar Cqs reported for the other markers.

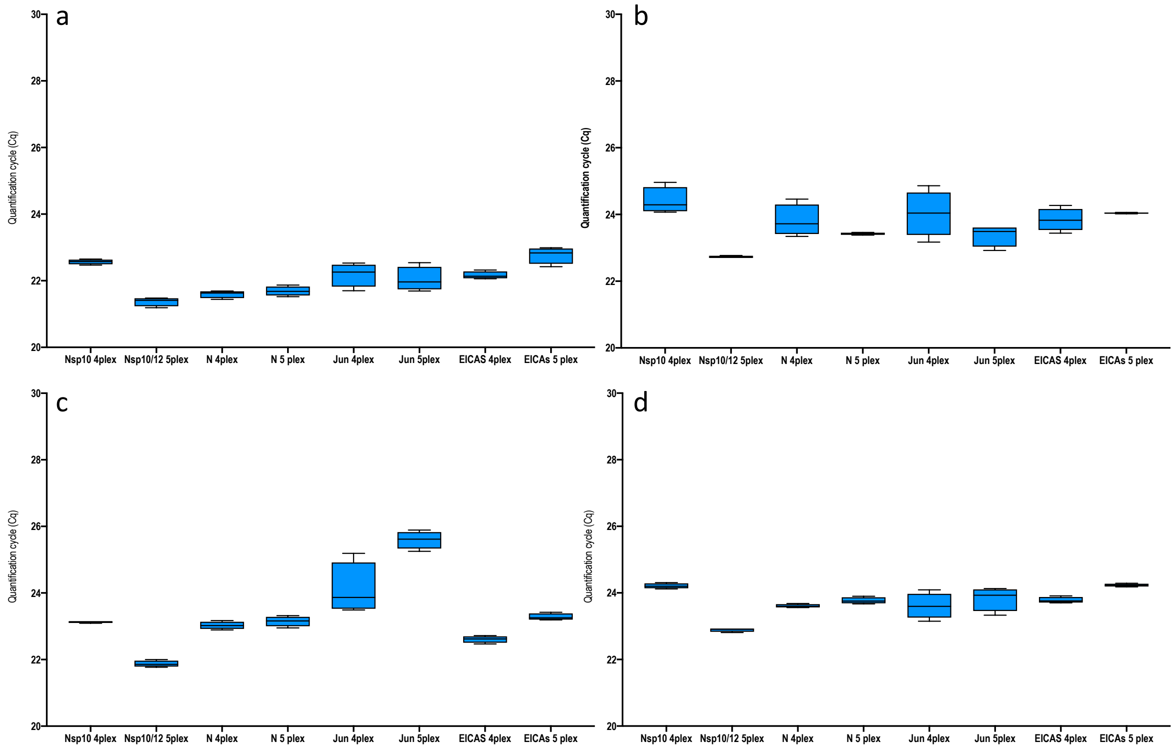

Figure S8. Comparison of 4 and 5plex assay with four RNAs extracted from clinical samples.

a. Sample C3. b. Sample C4. c. Sample C6. d. Sample C7.

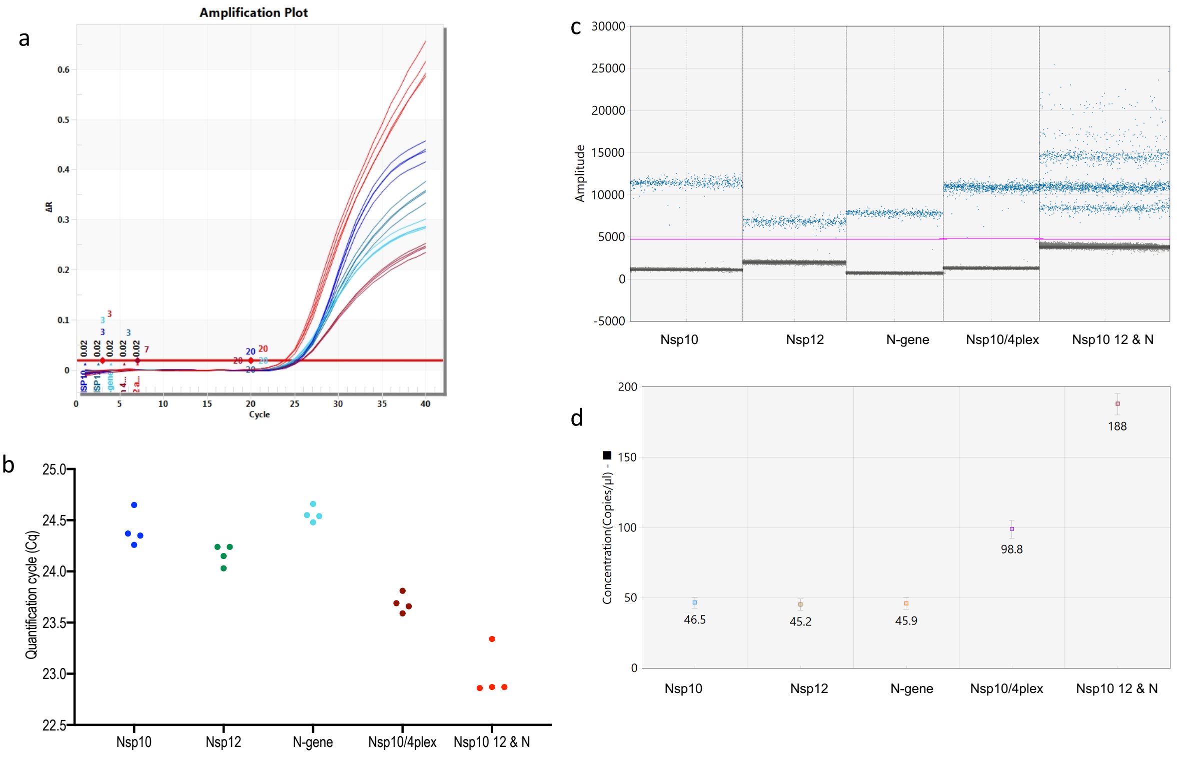

Figure S9. Performance of assay modified to consist of three FAM-labelled probes to detect SARS-CoV-2 targets. a. Amplification plots from four replicates in a qPCR assay. b. Plot of the Cqs recorded by the qPCR assay. c. ddPCR result showing the 1D amplitude of the same reactions recorded separately for Nsp10, Nsp12 and the N-gene, as part of the 4plex reaction (Nsp10) and for all three probes amplified in the same well. and 10 either in separate wells or together. d. Copy numbers of targets detected by Nsp10, Nsp 12, N-gene, the 4plex assay and all three probes calculated from the results shown above.

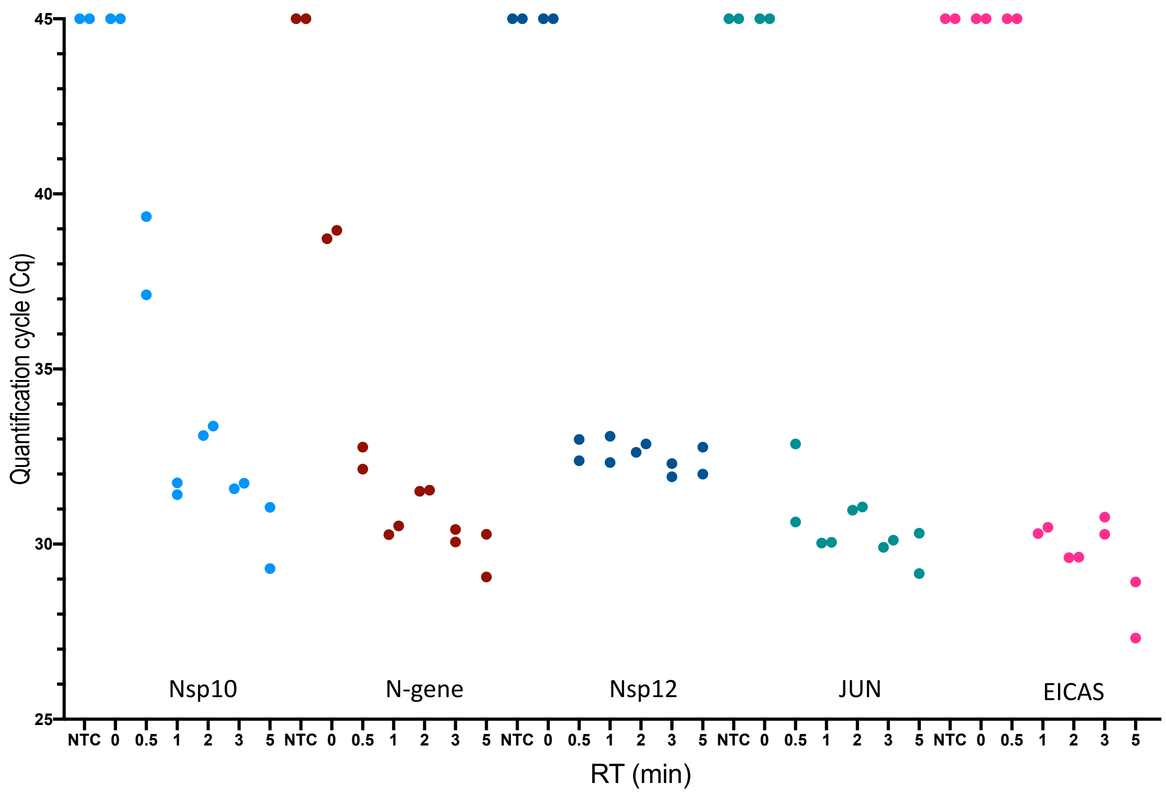

Figure S10. Effect of reducing RT times. The Cqs obtained for each of the five targets at the RT times are shown.

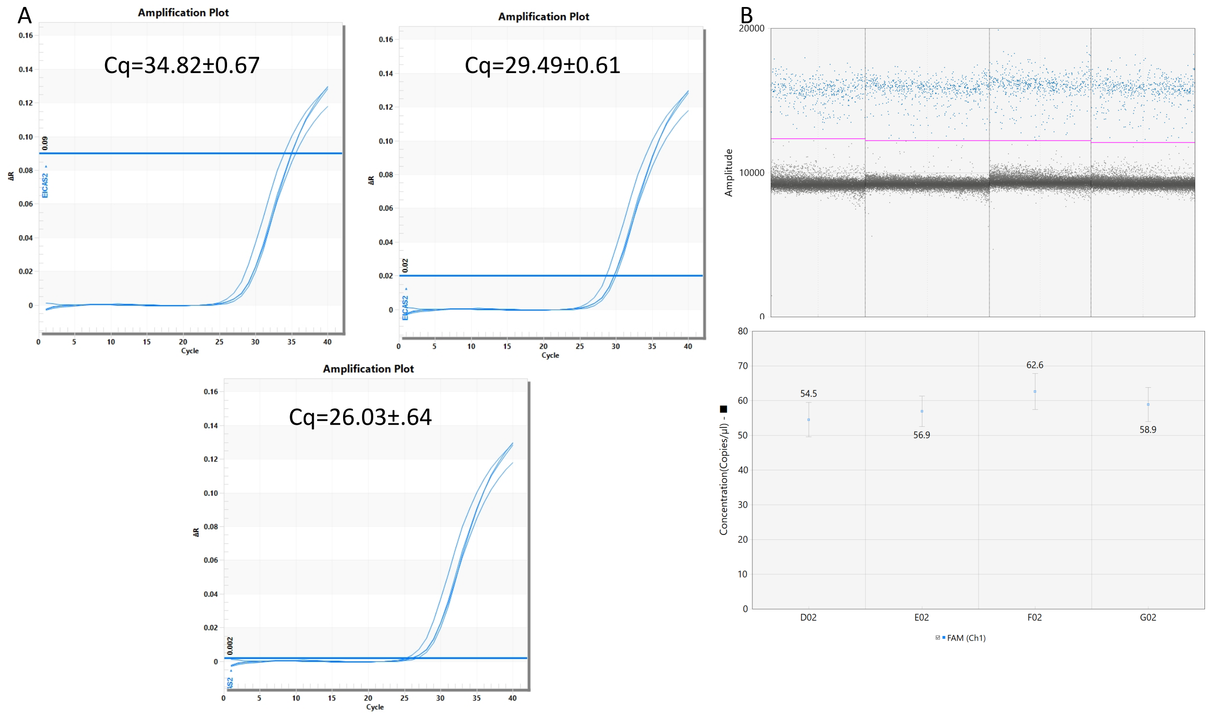

Figure S11. Quantification of viral load through EICAS. The same amount of EICAS was used in RT-qPCR and RT-ddPCR amplifications. a. Cq values obtained from qPCR experiments differ depending on where the threshold is set. b. Copy numbers calculated from ddPCR experiments are absolute values that can be used for quantification
